# To repair or replace‐the root dilemma in aortic dissections

**DOI:** 10.1111/jocs.16714

**Published:** 2022-06-23

**Authors:** Pradeep Narayan, Gianni D. Angelini

**Affiliations:** ^1^ Rabindranath Tagore International Institute of Cardiac Sciences, Narayana Health Kolkata India; ^2^ Bristol Heart Institute Bristol University Bristol UK

**Keywords:** aortic dissection, limited root repair, root replacement

## Abstract

Significant dilemma exists regarding the management of the aortic root pathology in acute aortic dissections. Several strategies for both repair and replacement exist and there is a lack of clarity on the superiority of one over the other. Important factors that influence management strategies include involvement of the sinuses, the competence of the aortic valve, the presence of Marfans syndrome, and connective tissue disorders, as well as availability of surgical expertise. The wide variability in these factors makes it unlikely for any one technique to be suitable for the management of all aortic roots pathology.

Management of the aortic root pathology in acute aortic dissection involving the thoracic aorta has been the subject of considerable interest and controversy and the study by Percy et al. examines the strategies for this clinically relevant issue in large nationwide analysis.^1^

Percy et al. in their study have divided these strategies broadly into two groups—those where the aortic valve was spared and those where it was replaced. However, the authors have not specified the various interventions that fall under these two groups. For instance, aortic valve replacement can be carried out separately with a supracoronary replacement of the ascending aorta as well as a composite root replacement. The former is a much simpler operation than a composite root replacement, and it would be important to note if these patients were treated as one and the same, as in both cases the aortic valve would be replaced. Similarly, the aortic valve repair group can potentially include valve resuspension as well as valve‐sparing root replacements, as in both cases the valve is spared but the two interventions are technically at the two ends of the surgical expertise spectrum. Thus, comparing outcomes under the headings of aortic valve repair or replacement may lead to outcomes that are hard to be generalized. A more clinically oriented way to group these patients would be to identify who require a root replacement and those who do not. This can be followed by an intragroup comparison of different techniques for root replacement and those where no root replacement or limited repair can be carried out.

A root replacement is indicated in the presence of gross dilatation or destruction of the sinuses of Valsalva, Marfans Syndrome, annuloaortic ectasia, or presence of intimal tear in the aortic root with or without the involvement of the coronary arteries.^2^, ^3^ The root replacement can be the more conventional composite root replacement (modified Bentall) with a mechanical valve in situ or in younger patients, with essentially normal aortic valves, the valve‐sparing techniques can be used.^4^, ^5^ The valve‐sparing root replacements can again be carried out using two different techniques, the aortic root remodeling technique^6^ or the reimplantation technique.^7^ Extensive comparisons have been drawn between the composite aortic root replacement and valve‐sparing root replacement for management of the aortic root that has resulted in several systematic reviews and meta‐analysis, often with conflicting observations.^8^, ^9^, ^10^, ^11^, ^12^ A valve‐sparing root replacement precludes the need to take oral anticoagulants, however, composite valve‐related complications have been found to be similar among the two strategies.^9^ Another study reported that the risk of endocarditis was lower with valve‐sparing techniques but it was associated with a higher rate of reoperations compared with composite root replacements.^11^ Others have reported lower incidence of thromboembolic events and similar durability of repair with both strategies.^8^

Repair techniques can include several surgical strategies where both the aortic valve and the sinuses (resuspension) are preserved, or the sinuses are partially replaced (Uni Yacoub procedure). When the aortic valves are normal, aortic regurgitation is essentially due to changes in the aortic root anatomy and can be easily addressed by valve resuspension which is a relatively simple, yet quite effective strategy.^13^ Valve resuspension is carried out in conjunction with the repair of the dissected aortic root and several techniques have been used to repair the dissected ascending aorta. This includes Teflon‐felt‐based repair techniques or glue‐based techniques either in isolation or in combination. The two well‐recognized Teflon‐based technique includes formation of the “neo‐media” and the “sandwich technique.” In the neo‐media technique, Teflon‐felt is inserted between the intima and the adventitia thus replacing the dissected media. The alternative technique is the sandwich technique where a Teflon‐felt strip is placed circumferentially along the inside and the outside of the aortic wall. Gelatin‐resorcinol‐formaldehyde‐(GRF) glue and Bioglue have been used along with Teflon‐felt repair as well as in isolation to approximate the aortic walls. The long‐term durability of the aortic root repair is a concern when GRF or Bioglue are used in isolation.^13^ Uni Yacoub is another repair technique where the dissection involves only the noncoronary sinus of Valsalva and limited excision of the sinus is performed.^5^ Depending on the repair technique there is significant variability in outcomes. While the freedom from reoperation with the “neo‐media” and the “sandwich” technique has been reported to be 89% and 79%, respectively, at 15 years,^14^, ^15^ the 10‐year freedom from reoperation with GRF alone is only 69%.^16^

The question whether limited root repair in aortic dissections is preferrable to root replacements has been examined by several studies. The discussion around the choice of technique mainly focusses on two considerations. First, does a more extensive root replacement leads to an increase in early mortality and in the longer term does it produce a more durable repair? If it leads to a more durable repair, can increased early mortality be an acceptable trade‐off? Proponents of aortic repair suggest that hospital mortality is of paramount importance and hence a more conservative repair may be preferrable in most cases.^4^, ^17^ However, it is increasingly been shown that more extensive root replacement techniques do not increase the risk of early mortality.^18^, ^19^, ^20^, ^21^ However, it must be borne in mind that most of these series are reported from high‐volume centers and whether the results seen in these studies are reproducible at all institutions remain questionable. On the question of the durability of repair while there is some evidence that root repair results in an increase in the risk of reoperation^20^ most studies show no difference in durability in the longer term^16^, ^18^, ^19^, ^21^ perhaps due to lower survival rates among these patients compared with age‐ and gender‐matched controls.^19^ Thus, it becomes obvious that in experienced hands root replacement does not pose any additional risk, however, there is also no overwhelming evidence of it being more durable than limited repair. So, experience with the technique may be the key for a successful short‐term outcome.

From a practical point of view the question that ought to be asked is—when to replace the aortic root in aortic dissection and when can we leave it alone. Younger age, intimal tear involving the aortic root with or without the involvement of the coronaries, dilated aortic root (>4.5 cm), and Marfans Syndrome are some of the indications where more aggressive root replacement is mandatory.^3^, ^16^, ^19^, ^21^ When a decision to replace the root has been taken the choice of the technique of root replacement composite or valve‐sparing, must be guided by the pathology and the surgical experience. If root replacement is not required, the next step is to assess if the aortic valve is incompetent and/or diseased and resuspension or a separate aortic valve replacement could be carried out along with supracoronary replacement of the ascending aorta.

The conflicting results with the same technique highlight the variabilities that exist in terms of the extent of aortic damage, the type of disease as well as the surgical expertise which may have a greater impact on the outcome rather than the technique itself. Percy et al. must be congratulated for carrying out this clinically relevant study and concluding that in selected cases' repair can be carried out. Management of the aortic root in aortic dissections is a complex problem and generalizing a treatment option of either aortic root replacement or repair would be erroneous. The management strategy must be individualized considering the patient, the pathology, and the surgeon expertise.

## CONFLICT OF INTEREST

The authors declare no conflict of interest.

## References

[1] Percy ED , Harloff MT , Newell PC , et al. Aortic root management in acute type a aortic dissection: a nationwide analysis. *J Card Surg*. 2022. 10.1111/jocs.16717 35870189

[2] Lai DT , Miller DC , Mitchell RS , et al. Acute type A aortic dissection complicated by aortic regurgitation: composite valve graft versus separate valve graft versus conservative valve repair. J Thorac Cardiovasc Surg. 2003;126(6):1978‐1986. 14688716 10.1016/s0022-5223(03)01279-0

[3] Ergin MA , McCullough J , Galla JD , Lansman SL , Griepp RB . Radical replacement of the aortic root in acute type A dissection: indications and outcome. Eur J Cardiothorac Surg. 1996;10(10):840‐844. 8911836 10.1016/s1010-7940(96)80308-3

[4] Westaby S , Saito S , Katsumata T . Acute type A dissection: conservative methods provide consistently low mortality. Ann Thorac Surg. 2002;73(3):707‐713. 11899170 10.1016/s0003-4975(01)03449-x

[5] El‐Hamamsy I , Ouzounian M , Demers P , et al. State‐of‐the‐art surgical management of acute type A aortic dissection. Can J Cardiol. 2016;32(1):100‐109. 26604123 10.1016/j.cjca.2015.07.736

[6] Yacoub MH , Gehle P , Chandrasekaran V , Birks EJ , Child A , Radley‐Smith R . Late results of a valve‐preserving operation in patients with aneurysms of the ascending aorta and root. J Thorac Cardiovasc Surg. 1998;115(5):1080‐1090. 9605078 10.1016/S0022-5223(98)70408-8

[7] David TE , Ivanov J , Armstrong S , Feindel CM , Webb GD . Aortic valve‐sparing operations in patients with aneurysms of the aortic root or ascending aorta. Ann Thorac Surg. 2002;74(5):S1758‐S1761. 12440659 10.1016/s0003-4975(02)04135-8

[8] Flynn CD , Tian DH , Wilson‐Smith A , et al. Systematic review and meta‐analysis of surgical outcomes in Marfan patients undergoing aortic root surgery by composite‐valve graft or valve sparing root replacement. Ann Cardiothorac Surg. 2017;6(6):570‐581. 29270369 10.21037/acs.2017.11.06 PMC5721109

[9] Benedetto U , Melina G , Takkenberg JJM , Roscitano A , Angeloni E , Sinatra R . Surgical management of aortic root disease in Marfan syndrome: a systematic review and meta‐analysis. Heart. 2011;15 97(12):955‐958. 21228428 10.1136/hrt.2010.210286

[10] Harky A , Fok M , Froghi S , Bilal H , Bashir M . Valve‐sparing aortic root repair compared to composite aortic root replacement: a systematic review and meta‐analysis. J Heart Valve Dis. 2017;26(6):632‐638. 30207112

[11] Soto ME , Ochoa‐Hein E , Anaya‐Ayala JE , Ayala‐Picazo M , Koretzky SG . Systematic review and meta‐analysis of aortic valve‐sparing surgery versus replacement surgery in ascending aortic aneurysms and dissection in patients with Marfan syndrome and other genetic connective tissue disorders. J Thorac Dis. 2021;13(8):4830‐4844. 34527322 10.21037/jtd-21-789 PMC8411183

[12] Hu R , Wang Z , Hu X , Wu H , Wu Z , Zhou Z . Surgical reconstruction of aortic root in Marfan syndrome patients: a systematic review. J Heart Valve Dis. 2014;23(4):473‐483. 25803974

[13] Leshnower BG , Chen EP . When and how to replace the aortic root in type A aortic dissection. Ann Cardiothorac Surg. 2016;5(4):377‐382. 27563551 10.21037/acs.2016.03.15 PMC4973127

[14] Rylski B , Bavaria JE , Milewski RK , et al. Long‐term results of neomedia sinus valsalva repair in 489 patients with type A aortic dissection. Ann Thorac Surg. 2014;98(2):582‐588. 24928674 10.1016/j.athoracsur.2014.04.050

[15] Mazzucotelli JP , Deleuze PH , Baufreton C , et al. Preservation of the aortic valve in acute aortic dissection: long‐term echocardiographic assessment and clinical outcome. Ann Thorac Surg. 1993;55(6):1513‐1517. 8512404 10.1016/0003-4975(93)91100-2

[16] Casselman FP , Tan ES , Vermeulen FE , Kelder JC , Morshuis WJ , Schepens MA . Durability of aortic valve preservation and root reconstruction in acute type A aortic dissection. Ann Thorac Surg. 2000;70(4):1227‐1233. 11081876 10.1016/s0003-4975(00)01966-4

[17] Schäfers HJ . Root replacement in acute dissection type A‐A superior procedure? J Thorac Cardiovasc Surg. 2018;155(1):8‐9. 28987739 10.1016/j.jtcvs.2017.09.038

[18] Di Eusanio M , Trimarchi S , Peterson MD , et al. Root replacement surgery versus more conservative management during type A acute aortic dissection repair. Ann Thorac Surg. 2014;98(6):2078‐2084. 25282163 10.1016/j.athoracsur.2014.06.070

[19] Yang B , Norton EL , Hobbs R , et al. Short‐ and long‐term outcomes of aortic root repair and replacement in patients undergoing acute type A aortic dissection repair: twenty‐year experience. J Thorac Cardiovasc Surg. 2019;157(6):2125‐2136. 30737109 10.1016/j.jtcvs.2018.09.129 PMC6588514

[20] Chiu P , Trojan J , Tsou S , Goldstone AB , Woo YJ , Fischbein MP . Limited root repair in acute type A aortic dissection is safe but results in increased risk of reoperation. J Thorac Cardiovasc Surg. 2018;155(1):1‐7.e1. 29042100 10.1016/j.jtcvs.2017.08.137 PMC5732846

[21] Wang Z , Greason KL , Pochettino A , et al. Long‐term outcomes of survival and freedom from reoperation on the aortic root or valve after surgery for acute ascending aorta dissection. J Thorac Cardiovasc Surg. 2014;148(5):2117‐2122. 24521975 10.1016/j.jtcvs.2013.12.059

